# Potential of rice tillering for sustainable food production

**DOI:** 10.1093/jxb/erad422

**Published:** 2023-11-02

**Authors:** Toshiyuki Takai

**Affiliations:** Japan International Research Center for Agricultural Sciences (JIRCAS), 305-8686 Tsukuba, Ibaraki, Japan; University of Sydney, Australia

**Keywords:** Climate change, food sustainability, grain yield, panicle number, quantitative trait locus (QTL), *Oryza sativa*, rice, strigolactones, tillering

## Abstract

Tillering, also known as shoot branching, is a fundamental trait for cereal crops such as rice to produce sufficient panicle numbers. Effective tillering that guarantees successful panicle production is essential for achieving high crop yields. Recent advances in molecular biology have revealed the mechanisms underlying rice tillering; however, in rice breeding and cultivation, there remain limited genes or alleles suitable for effective tillering and high yields. A recently identified quantitative trait locus (QTL) called *MORE PANICLES 3* (*MP3*) has been cloned as a single gene and shown to promote tillering and to moderately increase panicle number. This gene is an ortholog of the maize domestication gene *TB1*, and it has the potential to increase grain yield under ongoing climate change and in nutrient-poor environments. This review reconsiders the potential and importance of tillering for sustainable food production. Thus, I provide an overview of rice tiller development and the currently understood molecular mechanisms that underly it, focusing primarily on the biosynthesis and signaling of strigolactones, effective QTLs, and the importance of *MP3* (*TB1*). The possible future benefits in using promising QTLs such as *MP3* to explore agronomic solutions under ongoing climate change and in nutrient-poor environments are also highlighted.

## Teaser

Recent advances in the mechanisms underlying rice tiller development are reviewed, with a focus on the potential of utilizing tillering for sustainable food production under climate change.

## Introduction

At the beginning of crop domestication about 10 000 years ago, the global population was ~5 million and it has slowly increased since then until reaching 2 billion early in the 20th century (Evans, 1998). The Haber–Bosch process, first invented by Haber in 1908, involves the synthesis of ammonia from atmospheric nitrogen (N) and hydrogen (Haber, 1920). Haber’s discovery boosted the production of chemical fertilizer, thereby significantly increasing global agricultural productivity and aiding in the feeding of the growing global population (Erisman *et al.*, 2008). The global population was ~8 billion in 2022 and it is projected to continuously increase until it reaches 9.4–10.1 billion by 2050 (United Nations, 2022), indicating the need for increased food production. However, sustainable solutions must be pursued to address the challenges facing food production within the land that is currently cultivated, which encompasses both nutrient-rich and -poor soils, due to the limits on expansion of agricultural land and high population growth, particularly in sub-Saharan Africa where soil fertility is poor and most local farmers lack the financial means to access chemical fertilizers (Foley *et al.*, 2011; Saito *et al.*, 2019; Tsujimoto *et al.*, 2019).

Notably, population and economic growth have contributed to fossil-fuel combustion and deforestation, resulting in greenhouse gas emissions (e.g. CO_2_, CH_4_, and N_2_O) into the atmosphere and subsequent global warming (IPCC, 2014). Increasing global surface temperatures are projected to exacerbate climate and weather extremes, resulting in climate change that negatively affects crop production (Wheeler and von Braun, 2013; Anderegg *et al.*, 2020; IPCC, 2022). Therefore, developing genetically improved crops adapted to ongoing climate change and nutrient-poor environments is crucial for achieving sustainable food production and ensuring global food security in the future (Bailey-Serres, *et al.*, 2019; Acevedo *et al.*, 2020; Rivero *et al.*, 2021).

**Fig. 1.**
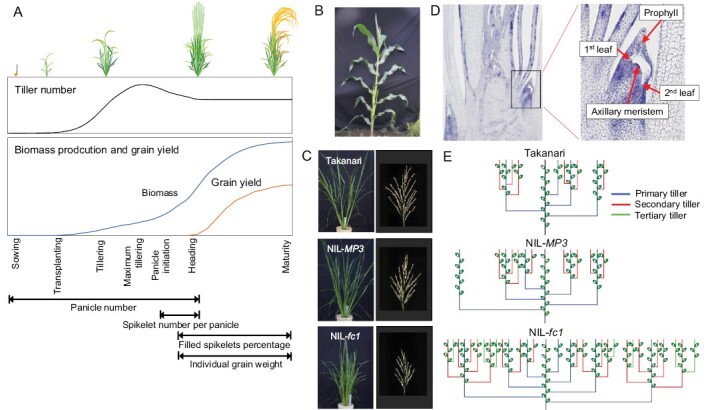
Development of tillers in rice. (A) Life cycle of rice plants. The periods when each yield component is determined are indicated below. (B) Plant architecture of maize. (C) Plant architecture and panicles in the rice cultivar Takanari, and the near-isogenic lines NIL-*MP3* and NIL-*fc1*. The images of panicles are from Takai *et al.* (2023) (published under creative commons BY-NC). (D) Longitudinal section of a young rice plant, with an axillary bud shown in detail. (E) Tiller development in Takanari, NIL-*MP3*, and NIL-*fc1* at the time of emergence of the 12th leaf.

Rice (*Oryza sativa*) is a staple food for over half of the world’s population and is grown in various different regions, from tropical to cold climates (Khush, 2005). Rice yield is a complex trait that is determined quantitatively by the yield components panicle number, spikelet number per panicle, filled spikelet percentage, and individual grain weight. Panicles are produced on productive tillers that develop over time from sowing to heading (Fig. 1A). Spikelets, the constituents of a panicle, are sink organs that absorb carbohydrates and begin to differentiate about 30 days before heading (Hoshikawa, 1975). The filled spikelet percentage and individual grain weight are attributable to photosynthetic assimilates translocated to the sink organs after heading (Ishii, 1995). Among these yield components, the panicle number is highly influenced by the environment because it takes the longest time to be determined (Fig. 1A).

The completion of the whole-genome sequencing of rice (International Rice Genome Sequencing Project, 2005) and subsequent progress in genomic technology have accelerated the resolution of quantitative trait loci (QTLs) into single genes involved in rice yield and its components, as well as elucidating the molecular mechanisms that underly them (Xing and Zhang, 2010; Miura *et al.*, 2011; Ikeda *et al.*, 2013). Although numerous QTLs have been identified and cloned for spikelet number per panicle and grain weight (size), effective QTLs that regulate the panicle number remain limited, probably due to factors such as trade-offs with other yield components and low heritability that may require more precise phenotyping for mapping and cloning (Yonezawa, 1997; Wang and Li, 2011). Notably, the causal genes identified for excessive branching phenotypes that led to the elucidation of the molecular mechanisms of rice tillering are undesirable for rice cultivation and breeding (Luo *et al.*, 2012). However, the recently identified QTL *MORE PANICLES 3* (*MP3*) can promote tillering and moderately increase the panicle number without negatively affecting spikelet number per panicle (Takai *et al.*, 2014, 2023) and can even enhance grain yield, as seen in a high-yielding elite cultivar grown in paddy fields under elevated atmospheric CO_2_ and nutrient-poor soils (Takai *et al.*, 2021, 2023). These findings emphasize that tillering and panicle number can play significant roles in achieving increased rice production under ongoing climate change and in nutrient-poor environments. More interestingly, *MP3* is a natural allele of *OsTB1/FC1* (*FINE CULM1*; Takeda *et al.*, 2003), an ortholog of the maize (*Zea mays*) domestication gene *Teosinte Branched 1* (*TB1*), which is involved in the repression of lateral branching and has significantly contributed to the change of maize plant architecture into a single stalk to increase yield (Doebley *et al.*, 1997). This again demonstrates the potential importance of *MP3* in modifying tiller/panicle numbers in rice breeding and cultivation, and it provides the incentive for this review of tiller formation and how such QTLs can be utilized to improve yields.

In this review, the architectures of maize and rice plants are introduced in the context of their relationships with *TB1* and *MP3*, respectively. This is followed by a brief overview of rice tiller development that highlights the importance of *MP3*. A detailed description of recent advances in the molecular mechanisms underlying rice tillering is then presented, with a focus on the biosynthesis and signaling of strigolactones, given that *MP3* acts downstream of their signaling pathway (Minakuchi *et al.*, 2010). An overview of promising QTLs related to tiller/panicle number for rice cultivation and breeding is provided next. Finally, ways in which QTLs such as *MP3* could facilitate improved tillering and panicle number to help adapt to or overcome ongoing climate change and nutrient-poor environments are proposed and discussed.

## Plant architecture in maize and rice

Modern-day maize plants typically have a single stalk with a few short branches, each of which is tipped by large, grain-bearing ears (Fig. 1B), whereas the plants of its wild ancestor, teosinte, have multiple long lateral branches on the main stalk, each tipped by a tassel and bearing many small ears at their nodes (Doebley, 2004). *TB1* is a gene that encodes a class II member of the TCP family of transcriptional regulators, and it represses the outgrowth of the axillary meristems and thus affects branch elongation (Doebley *et al.*, 2006). The architecture of maize plants was established by human selection for the highly expressed allele of *TB1* in southern Mexico during domestication about 9000 years ago (Doebley *et al.*, 1997; Matsuoka *et al.*, 2002; Studer *et al.*, 2011).

On the other hand, rice plants have natural variations in tiller/panicle number. Based on the number and size of the panicles produced, rice cultivars are broadly classified into panicle-number types (large number of panicles) and panicle-weight types (large-sized panicles) (Hanada, 1993). IR8 was the first high-yielding *indica* cultivar in the tropics and other subsequent IR series cultivars have followed it. They are all panicle-number types with a high tillering capacity and they contributed to the significant increase in rice productivity in Asia in the late 20th century, called the ‘Green Revolution’ (Peng *et al.*, 2008). Temperate *japonica* cultivars are adapted to and predominantly grown in Japan, Korea, and northern China. Around the same time as the ‘Green Revolution’, Japanese breeders also developed panicle-number type cultivars (Yonezawa, 1997; Yano *et al.*, 2019). Subsequently, panicle-weight types with large-sized panicles became the favoured plant architecture for increasing yield potential, as represented by new plant-type breeding in the Philippines (Peng and Khush, 2003), ‘super’ hybrid rice breeding in China (Peng *et al.*, 2008), and the super high-yielding rice project in Japan (Kumura, 1995; Yoshinaga *et al.*, 2013). These modern cultivars produce fewer panicles than the old panicle-number-type cultivars.

Takanari is a high-yielding *indica* cultivar in Japan with large-sized panicles and a functional allele of *OsTB1/FC1* (Fig. 1C). When this is replaced with a loss-of-function allele (*fc1*) in the Takanari genetic background, the subsequent near-isogenic line (NIL-*fc1*) produces 143% more panicles than Takanari but they are substantially smaller (Fig. 1C; Takai *et al.*, 2023). A trade-off between tiller/panicle number and spikelet number per panicle is often observed in rice plants (Wang and Li, 2011). In contrast, NIL-*MP3* in the same Takanari genetic background produces 28% more panicles and they have a similar size to Takanari (Fig. 1C; Takai *et al.*, 2023). *MP3* is a natural allele of *OsTB1/FC1* and is derived from Koshihikari, a leading temperate *japonica* cultivar in Japan. Three polymorphisms in the gene are considered to be the functional nucleotide polymorphisms in *MP3* (Takai *et al.*, 2023). How *MP3* increases the tiller/panicle number without decreasing the panicle size is considered in the next section, which provides an overview of the development of rice tillers.

## Development of tillers in rice

Tillers originate from axillary buds that develop at the axil of every leaf. When the leaf primordium of the mother stem differentiates at the shoot apical meristem, an axillary bud differentiates at 180° opposite to it (Hoshikawa, 1975). The axillary bud comprises an axillary meristem, a few leaf primordia, and a prophyll (Fig. 1D). After the bud completes its formation, it enters dormancy via apical dominance and inhibition of bud outgrowth by the shoot apex (Thimann and Skoog, 1934). Once apical dominance is released outgrowth begins, the first leaf derived from the axillary bud emerges from the subtending leaf sheath of the mother stem, and the bud develops as a tiller. Primary tillers refer to those that originate directly from the main stem, whilst secondary tillers arise from the primary tillers, and tertiary tillers develop from the secondary tillers.

Tiller development (tillering) and leaf emergence on the mother stem are closely linked. The ‘synchronously emerging characteristics of leaves and tillers’ is a relationship described by Katayama (1931), where the first leaf of the tiller emerges from the axil of the (*n*−3)th leaf when the (*n*)th leaf on the main stem emerges. Leaf–tiller synchronism is generally conserved in primary–secondary tillers and secondary–tertiary tillers. In theory, when the 12th leaf emerges from the main stem, the plant is expected to produce nine primary, 21 secondary, and 10 tertiary tillers. However, in reality, all the tillers might not be developed, as genetic and environmental factors regulate tillering. In terms of the genetic regulation by the alleles of *OsTB1/FC1*, Takai *et al.* (2023) observed a total of 10 tillers (four primary and six secondary) at the 12th main-stem leaf emergence in Takanari when plants were grown in pots in a greenhouse with nutrient-rich soil (Fig. 1E). Similarly, NIL-*MP3* produced 13 tillers (five primary and eight secondary), whereas NIL-*fc1* produced 31 tillers (seven primary, 17 secondary, and seven tertiary). Since higher-order tillers such as the tertiary ones emerge late and have restricted space, they are thinner and lighter and have smaller inflorescence meristems when compared with lower-order tillers. Thus, they form smaller panicles with fewer spikelets (Mu *et al.*, 2005; Yano *et al.*, 2019). This is one of the mechanisms underlying the trade-off between tiller/panicle number and spikelet number per panicle. Furthermore, the thinner and lighter tillers in NIL-*fc1* resulted in lodging occurring when plants were grown in paddy fields (Takai *et al.*, 2023). In contrast, no such negative effects were observed for the tiller characteristics of NIL-*MP3* and, as noted above, they had similar panicle size to Takanari. These findings indicate that a moderate increase in tiller/panicle number without the contribution of tertiary tillers is desirable to produce strong and productive tillers/panicles, and that *MP3* is the beneficial allele that enables it. In terms of environmental regulation, tillering is affected by plant spacing the availability of light and nutrients, and the conditions under which the plants are cultured (Yoshida, 1981). For example, when grown in pots with phosphorous (P)-deficient soil, Takanari and NIL-*MP3* produce only five and six tillers, respectively, at the 12th leaf emergence (Takai *et al.*, 2021), which are half the numbers observed when grown in nutrient-rich soil (see results quote above). Despite the significant decrease in tiller numbers in P-deficient soil, NIL-*MP3* still tended to produce more tillers than Takanari. The superiority of *MP3* in nutrient-poor soils is discussed below.

Rice plants typically show an increase in tiller number according to a sigmoid curve, reach a maximum, and then show a decrease as some tillers die (Fig. 1A). Surviving tillers, also known as productive tillers, can produce panicles. Productive tillers are derived from tillers that have more than two green leaves or are longer than two-thirds of the plant length at the maximum tillering stage (Matsushima, 1959). Unproductive tillers do not produce panicles and often die, and hence are considered at best as useless, or as competitive for resources such as solar energy, assimilates, and nutrients that are important for the productive tillers; thus, fewer unproductive tillers are desired in rice cultivation and breeding in order to achieve a high yield (Khush, 1995; Peng *et al.*, 1999; Ohe and Mimoto, 2002). Notably, the main stem is usually connected to the tillers through vascular bundles (Inosaka, 1958). Wang and Hanada (1982) reported the translocation of assimilates between the main stem and tillers at the 10th leaf age, whilst Nakamura (1959) observed the translocation of P from an unproductive tiller to a productive one at the heading stage, suggesting that unproductive tillers might contribute to the growth of productive tillers. Field experiments have supported this hypothesis, showing that decreasing the number of unproductive tillers by manual removal or by physically restricting tillering tends to decrease grain yield (Ao *et al.*, 2010). The increase in tillering associated with *MP3* also increases the number of unproductive tillers (Takai *et al.*, 2023), and hence further studies are required to quantify the contribution of unproductive tillers to grain yield.

## Molecular mechanisms underlying rice tillering

As described above, the formation and outgrowth of axillary buds regulate tiller development. It has been demonstrated that *OsTB1/FC1* works downstream of the strigolactone (SL) signaling pathway to inhibit the outgrowth of axillary buds (Minakuchi *et al.*, 2010), and this section describes the molecular mechanisms underlying the outgrowth of the buds, including the biosynthesis and signaling of SLs. First, an overview is given of the molecular network associated with axillary bud formation, and the section concludes with a consideration of the importance of *MP3* as the allele of *OsTB1/FC1* in terms of its role in the molecular networks.

**Fig. 2.**
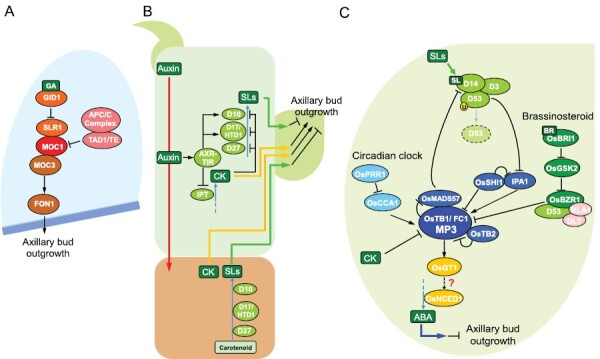
Current understanding of the molecular mechanisms of formation and outgrowth of axillary buds in rice. (A) Proposed model of axillary bud formation with a focus on MONOCULM1 (MOC1). GA, gibberellic acid. (B) Proposed model of the crosstalk among the phytohormones auxin, cytokinin (CK), and strigolactones (SLs) between shoots and roots. (C) Proposed model of the SL signaling pathway in axillary buds with a focus on OsTB1/FC1 (MP3). ABA, abscisic acid; BR, brassinosteroid. Lines ending with arrows and bars indicate promoting and inhibiting effects, respectively. See main text for details and abbreviations of proteins.

*MONOCULM1* (*MOC1*) was the first gene to be identified that controls rice tillering (Fig. 2A; Li *et al.*, 2003). *MOC1* encodes a GRAS family nuclear protein that is involved in the formation of axillary meristems. The *moc1* mutant lacks axillary buds, resulting in the development of only a main culm. LAX PANICLE 1 (LAX1) and LAX2 are required for the maintenance and formation of axillary meristems and they might interact with MOC1 (Komatsu *et al.*, 2003; Tabuchi *et al.*, 2011). Xu *et al.* (2012) and Lin *et al.* (2012) found that TILLERING AND DWARF 1/TILLER ENHANCER (TAD1/TE), a co-activator of the anaphase-promoting complex/cyclosome (APC/C) complex, degrades MOC1 in a cell cycle-dependent manner, resulting in decreased tiller numbers. In contrast, MOC1 is protected from degradation by physically binding to SLENDER RICE 1 (SLR1), a DELLA protein that acts as a repressor of gibberellin (GA) signaling (Liao *et al.*, 2019). GA is a phytohormone that controls diverse aspects of plant growth and development, including stem elongation (Yamaguchi, 2008). Interestingly, *SLR1* can inhibit stem elongation (Ikeda *et al.*, 2001) while increasing the tiller number by supporting *MOC1* (Liao *et al.*, 2019); this clearly provides a molecular explanation for the trade-off often observed between plant height and tiller number. Moreover, the physical interaction between MOC1 and MOC3, the rice ortholog of Arabidopsis WUSCHEL (WUS; Lu *et al.*, 2015; Tanaka *et al.*, 2015), can up-regulate the expression of *FLORAL ORGAN NUMBER1* (*FON1*) that encodes the rice ortholog of Arabidopsis CLAVATA1 (CLV1; Suzaki *et al.*, 2004), leading to enhanced tiller bud outgrowth and thus tiller number (Shao *et al.*, 2019). Similar to the *moc1* mutant, the *moc3* mutant cannot form normal axillary meristems, whereas the *fon1* mutant forms them normally but fails to elongate tiller buds. This indicates that MOC3 is involved in the formation of axillary buds while FON1 specifically affects their outgrowth (Shao *et al.*, 2019). The coordinated regulation by MOC1, MOC3, and FON1 reveals a direct connection from the formation of the tiller bud to its outgrowth, although events downstream of FON1 remain unknown.

The phenomenon of axillary bud outgrowth, which is controlled by apical dominance, has been the subject of research for over a century in numerous plant species. Auxins, which are a class of plant growth hormones that are mainly synthesized in young leaves located at the shoot apex (Ljung, *et al.*, 2001), are transported basipetally to roots in the polar auxin transport stream and they inhibit axillary bud outgrowth (Snow, 1929; Thimann and Skoog, 1934), although they do not migrate to axillary buds (Fig. 2B) (Booker *et al.*, 2003). In contrast, cytokinins (CKs), a class of plant hormones that are synthesized in shoots (Nordström *et al.*, 2004) and roots (Chen *et al.*, 1985), are transported acropetally in the xylem, and enter axillary buds and promote their outgrowth (Fig. 2B) (Wickson and Thimann, 1958; Sachs and Thimann, 1967; Müller and Leyser, 2010).

Recent advances in molecular biology and the discovery of SLs, a group of terpenoid plant hormones (Gomez-Roldan, *et al.*, 2008; Umehara *et al.*, 2008), have facilitated the understanding of the molecular mechanisms underlying apical dominance and hence rice tillering. SLs are synthesized in roots and shoots, transported acropetally in the xylem, and move to axillary buds and inhibit their outgrowth (Fig. 2B) (Domagalska and Leyser, 2011; Kameoka and Kyozuka, 2018). In rice, DWARF27 (D27; Lin *et al.*, 2009), D17/HIGH-TILLERING DWARF1 (HTD1; Zou *et al.*, 2006), and D10 (Arite *et al.*, 2007) are proteins that act as enzymes in SL biosynthesis. Hayward *et al.* (2009) revealed that auxin up-regulates the expression of Arabidopsis *MAX3* and *MAX4*, the orthologs of *D17/HTD1* and *D10*, respectively, via AUXIN RESISTANT1–TRANSPORT INHIBITOR RESPONSE1 (AXR1–TIR1)-mediated degradation of the auxin/IAA protein in shoots. A similar up-regulation has been observed in *D10* in shoots of rice when auxin is applied exogenously (Arite *et al.*, 2007), indicating that auxin increases SL biosynthesis in shoots. On the other hand, Tanaka *et al.* (2006) observed that auxin down-regulates the expression of pea *Isopentenyltransferase* (*IPT*) family genes in the nodal stem, which encode a key enzyme in CK biosynthesis, through the AXR–TIR-dependent auxin signaling pathway. A similar suppression has been observed in *IPT* genes in rice after auxin treatment (Minakuchi *et al.*, 2010). Exogenous CK analogs can also repress the expression of *D27*, *D17/HTD1*, and *D10* at the nodes in rice (Xu *et al.*, 2015). Overall, these results clearly show that crosstalk among auxin, SLs, and CK in shoots regulates axillary bud outgrowth, with auxin inhibiting CK biosynthesis but promoting SL biosynthesis, while CK inhibits SL biosynthesis (Fig. 2B) (Beveridge and Kyozuka, 2010).

Upon entering the axillary buds, SLs are perceived by the D14 protein (Fig. 2C) (Nakamura *et al.*, 2013). This belongs to the α/β-fold hydrolase superfamily (Arite *et al.*, 2007) and is thought to be transported to axillary buds via the phloem (Kameoka *et al.*, 2016). Various models have been proposed to explain how D14 transforms into an active signaling state and perceives SLs, but they are still under debate (Yao *et al.*, 2016; Shabek *et al.*, 2018; Seto *et al.*, 2019; Mashiguchi *et al.*, 2021). Other important actors in the SL signaling pathway are D3, an F-box protein (Ishikawa *et al.*, 2005), and D53, a Clp ATPase protein (Jiang *et al.*, 2013; Zhou *et al.*, 2013). D53 inhibits the downstream events of SL signaling in the absence of SLs (Jiang *et al.*, 2013; Zhou *et al.*, 2013). The reception of SLs by D14 triggers the formation of a protein complex of D14, D3, and D53, resulting in ubiquitination and degradation of D53 via the 26S proteasome pathway (Jiang *et al.*, 2013; Zhou *et al.*, 2013). D53 degradation triggers the downstream SL signaling: IDEAL PLANT ARCHITECTURE 1 (IPA1; Jiao *et al.*, 2010; Miura *et al.*, 2010) is induced as a transcriptional activator (Song *et al.*, 2017) and promotes expression of *OsTB1/FC1* (Fig. 2C) (Lu *et al.*, 2013). As previously described, *OsTB1/FC1*, which includes the *MP3* allele, is a negative regulator that suppresses axillary bud outgrowth (Takeda *et al.*, 2003; Takai *et al.*, 2023). Upon up-regulation of *OsTB1/FC1*, its protein enhances the expression of *GRASSY TILLER1* (*GT1*) by directly binding to its promoter (Kumar *et al.*, 2021). OsGT1 is likely to regulate genes such as *9-CIS-EPOXYCAROTENOID DIOXYGENASE 1* (Os*NCED1*) that encode abscisic acid (ABA) biosynthesis enzymes, which are involved in axillary bud dormancy and hence suppression of axillary bud outgrowth in rice (Luo *et al.*, 2019).

While *OsTB1/FC1* expression is promoted by IPA1, Guo *et al.* (2013) determined that the OsTB1/FC1 protein can interact with the OsMADS57 protein that suppresses *D14* expression. The reduction in OsMADS57 activity thereby reduces the inhibition of *D14* and thus establishes a positive feedback loop among the D14–D3–D53 complex, *IPA1*, and *OsTB1/FC1* (Fig. 2C). Furthermore, recent studies have revealed that *OsTB1/FC1* is involved in other rice signaling pathways in addition to SL. F. Wang *et al.* (2020) showed that *CIRCADIAN CLOCK ASSOCIATED1* (*OsCCA1*), positively controls *OsTB1/FC1* expression. Conversely, *OsTB1/FC1* expression is down-regulated by SHORT INTERNODES1 (SHI1; Duan *et al.*, 2019) and the D53– BRASSINAZOLE RESISTANT 1 (OsBZR1) complex (Fang *et al.*, 2020) as well as by CK (Minakuchi *et al.*, 2010). SHI1 is a plant-specific transcription factor of the SHI family in rice that interacts with IPA1 to inhibit its function (Duan *et al.*, 2019). OsBZR1 functions in the brassinosteroid (BR) signaling pathway (Bai *et al.*, 2007) and forms a D53–OsBZR1 complex in rice with REDUCED LEAF ANGLE1 (RLA1) and DWARF AND LOW-TILLERING (DLT) to suppress *OsTB1/FC1* in the presence of BR (Fang *et al.*, 2020). Moreover, the function of OsTB1/FC1 is also suppressed by physical interaction with OsTB2, a homolog of OsTB1/FC1 (Lyu *et al.*, 2020). These findings indicate that *OsTB1/FC1* functions as a key integrator of multiple signaling pathways to balance rice tillering (Minakuchi *et al.*, 2010; Wang *et al.*, 2018). The importance of the balancing of this gene is also evident from the fact noted above that NIL-*fc1* plants (in which the function in regulating tillering is lost) exhibit an abnormal phenotype of extremely high branching. In this case, the three polymorphisms in *MP3* might have the well-executed effect of partially decreasing the function of OsTB1/FC1 to moderately increase tiller/panicle numbers.

## Effective QTLs for tiller/panicle number to increase grain yield

As described above, many genes for controlling rice tillering have been identified; however, most studies have used mutant lines with excessive branching phenotypes, which is not applicable to rice cultivation and breeding (Luo *et al.*, 2012). Therefore, increases in rice productivity require effective genes or alleles. Some promising QTLs associated with tiller or panicle number have recently been cloned as a single genes, including *MP3* (Zhang *et al.*, 2017; Y. Wang *et al.*, 2020; Liu *et al.*, 2021; Takai *et al.*, 2023).

*qWS8/ipa1-2D*, is a novel allele of the SL-signaling gene *IPA1*, and it generates large panicles and a moderate panicle number, resulting in increased grain yield (Zhang *et al.*, 2017). A NIL with a partial loss-of-function allele of the SL biosynthesis gene *D17/HTD1* also produce more tillers (~20%) than the parental cultivar with a functional allele and have increased grain yield (Y. Wang *et al.*, 2020). The beneficial alleles of these two genes appear to be useful for future breeding programs, and indeed the allele of *D17/HTD1* has already been utilized in modern, high-yielding rice cultivars since the Green Revolution in the 1960s, including IR8 (Y. Wang *et al.*, 2020). However, *IPA1* causes a notable trade-off between the panicle number and spikelet number per panicle and dramatically changes the plant architecture, which perhaps makes it harder for farmers to manage plants in paddy fields.

Using a genome-wide association study, Liu *et al.* (2021) have identified *OsTCP19*, a negative regulator of rice tillering, and its beneficial allele increases the tiller number and grain yield in response to N application. This allele has largely been lost in modern rice cultivars, and its introduction might be of benefit in recent high-yielding cultivars. Finally, as described above, *MP3* is a natural allele of *OsTB1/FC1* and moderately promotes tillering, increasing the panicle number by 20–30% and the spikelet number per unit area by 20% without negatively affecting panicle and tiller sizes (Takai *et al.*, 2023). The *MP3* allele can be observed in most accessions in the temperate *japonica* subgroups but is rarely found in the *indica* subgroup. More interestingly, *MP3* (*OsTB1/FC1*) has not been involved and utilized in artificial selection during domestication or breeding, in contrast to maize *TB1*, although the reason is unclear (Xu *et al.*, 2011; Takai *et al.*, 2023). Nevertheless, a clear functional differentiation of the *MP3* allele between the temperate *japonica* and *indica* subgroups indicates that it could be useful in improving *indica* cultivars. A free-air CO_2_ enrichment experiment (579 μmol mol^−1^) has revealed a significant increase in grain yield in NIL-*MP3* compared to the parental cultivar Takanari by filling the increased spikelet number at elevated CO_2_. Based on these results, Takai *et al.* (2023) concluded that *MP3* could contribute to an increase in rice production under climate change with rising atmospheric CO_2_.

## Potential of *MP3* for sustainable food production

The moderate promotion of tillering by *MP3* notably enhances grain yield at an elevated CO_2_ concentration projected to be reached this century. *MP3* has potential for benefitting various aspects of cropping and it could be an effective genetic factor for sustainable production in rice and other crops. This section considers possible future benefits in utilizing *MP3* for agronomic solutions under ongoing climate change and in nutrient-poor environments.

**Fig. 3.**
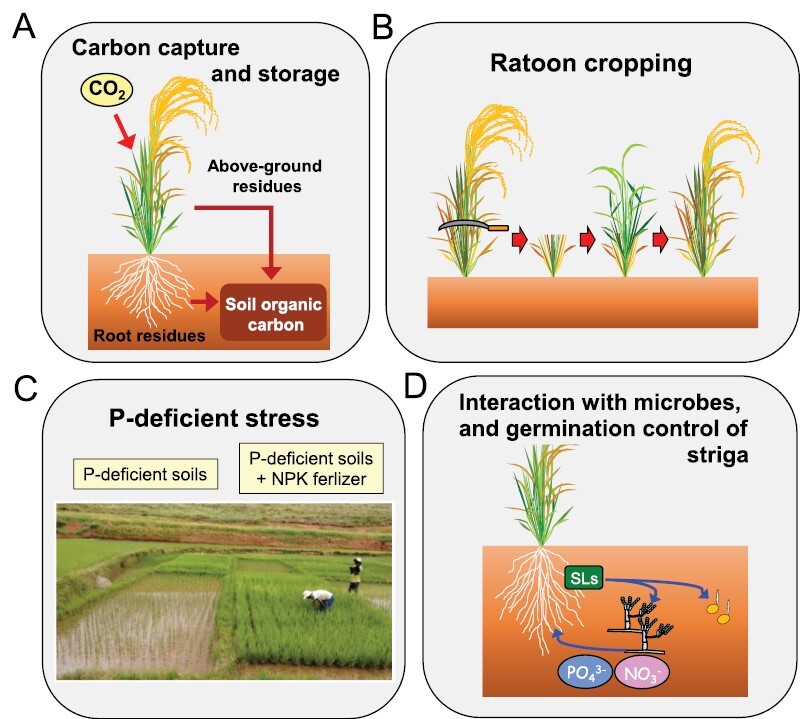
Potential future benefits of utilizing *MP3* for sustainable food production. (A) Increased carbon capture in above- and below-ground biomass, and increases in soil organic carbon. (B) Improved yields in ratoon cropping systems. (C) Improved crop performance in nutrient-poor soils, particularly under phosphorous deficiency. (D) Improved interactions with arbuscular mycorrhizal fungi, and possible reduction in stimulation of germination of seeds of parasitic plants.

First, *MP3* could mitigate rising atmospheric CO_2_ levels by increasing carbon-capture from the atmosphere and promoting the storage of soil organic carbon (SOC; Fig. 3A). Studies have demonstrated that major C_3_ cereals produce 10–20% more above-ground biomass at elevated CO_2_ levels (550–600 μmol mol^−1^) than at current ambient levels (380–400 μmol mol^−1^) (Long *et al.*, 2004; Hasegawa *et al.*, 2013). Interestingly, *MP3* further enhances the production of above-ground biomass by 3.5% at elevated CO_2_ due to the greater sink capacity, leading to an increase in carbon capture (Takai *et al.*, 2023). Notably, elevated CO_2_ stimulates root growth and thus root biomass, resulting in increased C input to the soil (Nie *et al.*, 2013), which generally induces increased storage of SOC until decomposing microorganisms release it (Olson, 1963; Shirato, 2020). Although further studies are required to determine whether *MP3* enhances root biomass at elevated CO_2_, Terrer *et al.* (2021) have reported that SOC can accumulate as elevated CO_2_ increases above-ground biomass in grasslands. Given that the global area planted with rice was 164 million ha in 2020 (see https://www.fao.org/faostat/en/#home), the enhancement of biomass production by *MP3* has the potential to substantially increase both land CO_2_ uptake and SOC storage, and thus ultimately help to alleviate increasing atmospheric CO_2_ levels (Weigmann, 2019).

Second, *MP3* might be advantageous in the ratoon cropping of rice, which is the practice of harvesting a second crop from the stubble of the first crop (Fig. 3B; Plucknett *et al.*, 1970). Although this cropping system has a long history of being studied in India, the USA, and China, particularly during the 1950s and 1960s (Roy, 1959; Jones, 1993), it has not been widely adopted by farmers due to a lack of suitable rice cultivars, unstable yield, and incompletely established management practices (Plucknett *et al.*, 1970; Yuan *et al.*, 2019). However, it has recently been reconsidered owing to its advantages in terms of reduced labor costs (no need for transplanting a second crop), less water use, and less potential for greenhouse gas emissions than double-cropping of rice due to its shorter growth period (Jones, 1993; Yuan *et al.*, 2019; Song *et al.*, 2022). Generally, the grain yield of the ratoon crop is only 28.6–64.3% of that of the first crop (W. Wang *et al.*, 2020). In terms of increasing grain yield in ratoon crops, many studies have indicated the importance of a higher non-structural carbohydrate (NSC) content and a higher leaf area index (LAI) in the stubble at the harvest of the first crop (Turner and Jund, 1993; He *et al.*, 2019; Nakano *et al.*, 2020, 2021; Tanaka *et al.*, 2022). A high cutting height of the first crop maintains high LAI and a large amount of NSC in the stubble, which are used as sources to promote axillary bud outgrowth after the removal of apical dominance caused by cutting the shoots above (Nakano *et al.*, 2020; W. Wang *et al.*, 2020). Because the increase in grain yield in ratoon cropping is attributable to improved panicle number rather than spikelet number per panicle (Nakano *et al.*, 2020, 2022), the promotion of axillary bud outgrowth by *MP3* could enhance grain yield and be useful in the ratoon cropping system, since the genetic factors that directly control ratooning ability remain unidentified (W. Wang *et al.*, 2020).

Third, *MP3* may be effective in increasing rice productivity in sub-Saharan Africa (SSA), where most paddy fields are characterized as nutrient-poor soils (Fig. 3C). Large areas of SSA are deficient in the major nutrients N and P (Saito *et al.*, 2019). Even when P is present in the soil, it is largely fixed by active Al and Fe, rendering it unavailable for absorption by plants (Nishigaki *et al.*, 2019). Inadequate amounts of N and in particular P significantly restrict rice tillering, resulting in reduced panicle numbers and hence a decrease in grain yield (Dobermann and Fairhurst, 2000). Although the application of chemical fertilizers can improve soil nutrient levels, phosphate rock, the source of P fertilizer, is a finite and non-renewable resource (Cordell *et al.*, 2009). Soil erosion induced by increased precipitation caused by global warming can result in substantial losses of soil P (Alewell *et al.*, 2020). Furthermore, local SSA farmers lack the capacity to purchase sufficient fertilizers (Vanlauwe *et al.*, 2014). Therefore, developing application techniques that require the least amount of fertilizer (Tsujimoto, *et al.*, 2019) as well as genetically improving rice varieties to have high nutrient usage efficiencies and/or vigorous growth under nutrient-poor soils is necessary (Ismail *et al.*, 2007; Tsujimoto *et al.*, 2020). Dipping seedling roots in P-enriched slurry, called ‘P-dipping’, is considered a promising and cost-effective technique in fertilizer management for improving usage and rice productivity (De Datta *et al.*, 1990; Rakotoarisoa *et al.*, 2020). Recently, we tested the performance of NIL-*MP3* in nutrient-poor soils in the central highland of Madagascar and found that it promoted tillering and produced 19% more panicles and 12% more spikelets per m^2^ than Takanari, with the exception of fields with severe P deficiency (Takai *et al.*, 2021). The results indicated that the effect of *MP3* on tillering considerably surpasses the restriction of tillering by P-deficient soils. Accordingly, we are currently introducing *MP3* into a leading Madagascan cultivar, X265 (Diagne *et al.*, 2015) using backcrossing and marker-assisted selection to boost rice productivity in nutrient-poor soils.

Finally, *MP3* could be useful in realizing more sustainable and environmentally friendly agriculture through interactions with microorganisms and controlling germination of parasitic weeds (Fig. 3D). As described above, *MP3* is a natural allele of *OsTB1/FC1* that works downstream of the SL signaling pathway (Takai *et al.*, 2023). In addition to their role as endogenous inhibitors of shoot branching, SLs act as symbiotic signals with arbuscular mycorrhizal (AM) fungi to acquire inorganic nutrients such as P and N, particularly in nutrient-poor soils (Akiyama *et al.*, 2005; Umehara *et al.*, 2010). SLs exudated from the roots of host plants induce the hyphal branching of AM fungi in the rhizosphere. Developed hyphae reach the root surface of the host plants, produce hyphopodia on the root epidermis, penetrate the roots, form arbuscules in the roots, and complete root colonization (Bonfante and Genre, 2010). Interestingly, recent studies have revealed strong defects in arbuscule formation in *d3* mutants that are defective in SL signaling (Yoshida *et al.*, 2012), and weak formation of hyphopodia in *d10* and *d17* mutants that are defective in SL biosynthesis (Kobae *et al.*, 2018), indicating that some genes of SL biosynthesis and signaling are involved in AM fungal colonization. These results suggest that *MP3* can affect the mycorrhization of AM fungi. Unlike the loss-of-functional alleles in these mutants, *MP3* is a functional allele and thus could be valuable in breeding cultivars with improved nutrient acquisition through enhanced symbiosis with AM fungi (Chesterfield *et al.*, 2020). Furthermore, SLs act as seed-germination stimulants of root parasitic plants such as *Striga* and *Orobanche* species (Bouwmeester *et al.*, 2003). Whilst ‘suicide germination’ induced by the application of SL agonists in the absence of host plants is a promising way to reduce *Striga* parasitism (Uraguchi *et al.*, 2018), a decrease in SLs released from host plant roots is another important way to help overcome parasitic plants. If the natural variations in *OsTB1/FC1* including *MP3* influence the amounts of SLs in the root exudates, then the allele that results in lower SLs could be useful in controlling parasitic weeds.

## Conclusions

Tillering is a fundamental trait in rice for ensuring a sufficient panicle number and sink size (total spikelet number per unit land area). While efforts are ongoing to improve sustainable crop production in a changing climate by enhancing the ability of plants to produce photosynthetic assimilates such as sugars (Balley-Serres *et al.*, 2019), research on *MP3* has demonstrated the importance of improving the panicle number and sink size to enhance grain yield at elevated atmospheric CO_2_. While both improving the spikelet number per panicle and the panicle number can increase sink size, this review has demonstrated that it is improving the panicle number that might have broader applicability in rice cultivation because it is more beneficial in scenarios such as ratoon cropping and nutrient-poor soils. Due to ongoing climate change, drought and rising temperatures are significant challenges to sustainable crop production. Just as a single stalk is the plant architecture associated with *TB1* in maize, upland rice has a low tiller/panicle number probably as an adaptation to limited water availability (Uddin and Fukuta, 2020); however, high yields cannot be expected with a low tiller/panicle numbers. A combination of high tiller/panicle numbers with deeper rooting might be able to mitigate the challenge caused by drought and lead to high-yield performance (Uga *et al.*, 2013). Regarding rising temperatures, while the effects on tillering and panicle numbers require further elucidation, the negative relationship observed between night temperatures and panicle numbers suggests the importance of increasing tiller/panicle numbers under global warming (Peng *et al.*, 2004). Finally, as described in this review, the formation of rice tillers is affected by various factors including heredity, plant hormones, and cultivation environments. Although exogenous application of plant hormones or chemical fertilizers can also alter tillering, this review has demonstrated that inherited genetic regulation is a low-cost and robust option to artificially regulate the tiller/panicle number in rice cultivation, just as humans have brought about the change from teosinte to maize through *TB1* during the process of domestication. Therefore, it is proposed that *MP3* and its homologs possess significant potential as genetic factors/regulators for ensuring sustainable production in rice and other crops.
